# Exploring the Bioaccessibility of Roasted Japanese Green Tea: Impact of Simulated Gastrointestinal Digestion

**DOI:** 10.3390/foods14020311

**Published:** 2025-01-17

**Authors:** Wei Qin, Sunantha Ketnawa

**Affiliations:** 1 Faculty of Health Sciences, Hokkaido University, Kita-12, Nishi-5, Kita-Ku, Sapporo 060-0812, Japan; 2 Graduate School of Horticulture, Chiba University, 648, Matsudo, Matsudo 271-8510, Japan; sunantha.ketnawa@gmail.com; 3 Food Science and Technology Program, School of Agro-Industry, Mae Fah Luang University, Chiang Rai 57100, Thailand

**Keywords:** roasted green tea, bioactive compounds, antioxidant, in vitro digestion, bioaccessibility

## Abstract

In this study, the effects were explored of digestive enzymes and pH on the bioaccessibility of polyphenols, flavonoids, and antioxidant activities in Hojicha (roasted green tea, RT) infusions during simulated in vitro digestion. Roasting modifies its polyphenolic profile and reduces bitterness, making it a popular variation of green tea. In this study, RT was used for assessing how the roasting-induced changes influenced the tea’s bioaccessibility and stability under digestive conditions. A two-step gastrointestinal digestion model was applied to mimic real digestion. Total polyphenol content (TPC), total flavonoid content (TFC), and antioxidant activity (DPPH, ABTS, FRAP, and MIC) were measured at different digestion stages. Gastric conditions led to a 2.07-fold reduction in TPC and a 4.27-fold reduction in TFC. Digestive enzymes enhanced bioactive compound stability, with TPC and TFC bioaccessibility reaching 56% and 25% in the simulated digestion with digestive enzymes (MD) group, compared to 52% and 20% in the without digestive enzymes (WOE) group. Antioxidant activities were also better preserved, with antioxidant activity retention at 31% in the MD samples versus 19% in the WOE. These findings emphasize the key role of digestive enzymes in maintaining the antioxidant potential of roasted green tea during digestion, providing insight into future research on roasting methods and tea functionality for product development.

## 1. Introduction

Green tea has long been recognized for its rich content of bioactive compounds, particularly polyphenols such as catechins, which are known for their antioxidant, anti-inflammatory, and potential health-promoting effects [1,2,3]. Roasted Japanese green tea (RT), commonly referred to as “Hojicha”, is a widely consumed variant of green tea that undergoes a roasting process at high temperatures (160 °C to 180 °C) [4]. This process not only alters the flavor profile of the tea but also impacts its chemical composition, particularly its polyphenol content [5,6,7]. Roasting enhances the tea’s caramel and nutty aroma while reducing the astringency and bitterness associated with catechins [5,8,9,10,11], making it more palatable to a broader range of consumers.

Hojicha has become increasingly popular due to its milder flavor and reduced caffeine content, which make it suitable for consumption at any time of day, including by individuals who are sensitive to caffeine [5,8]. The roasting process also contributes to the development of specific volatile compounds, such as pyrazines, pyrroles, and furans, which provide Hojicha with its distinctive aroma [12,13,14]. However, high roasting temperatures can cause the thermal degradation and transformation of bioactive compounds, such as catechins and flavonoids, which may influence their stability and bioaccessibility during digestion. For example, catechins may undergo oligomerization under roasting conditions, leading to the formation of new polyphenolic compounds with potentially altered functional properties [5,15]. These transformations are critical to the development of roasted tea’s flavor but may also impact the stability and bioaccessibility of these compounds during digestion. Similar effects have been observed in other thermally processed foods. For example, in cocoa, high-temperature roasting degrades certain flavan-3-ols while enhancing the formation of stable derivatives, which can influence their functional properties [6]. In coffee, polyphenols can bind to Maillard reaction products, affecting their solubility and bioaccessibility [7]. These findings highlight that thermal processing not only modifies the chemical composition but may also influence the digestive behavior of bioactive compounds.

While studies have explored the sensory properties, volatile profiles, and dry leaf composition of roasted green tea [5,8,15], there remains limited information regarding the bioaccessibility of its bioactive compounds during digestion. Bioaccessibility refers to the fraction of bioactive compounds released from the food matrix during digestion and made available for potential absorption in the gastrointestinal tract [16,17]. This parameter is crucial for understanding the potential health benefits of food products, particularly for polyphenols, whose stability and solubility can be significantly influenced by digestive conditions. Understanding the bioaccessibility of polyphenols and the antioxidant activities of roasted green tea during digestion is essential to better evaluate its functional properties as a health-promoting beverage.

In this study, the hypothesis is that the gastrointestinal digestion process significantly influences the bioaccessibility of bioactive compounds in roasted Japanese green tea, with digestive enzymes enhancing the stability of polyphenols and antioxidant activities under simulated gastric and intestinal conditions. To test this hypothesis, we investigate changes in total polyphenol content (TPC), total flavonoid content (TFC), and antioxidant activities of roasted green tea using assays such as DPPH, ABTS radical scavenging activity, ferric-reducing antioxidant power (FRAP), and metal ion chelating (MIC) activity. By examining the impact of digestion on these parameters, in this study, the aim is to provide insights into the health potential of roasted green tea as a functional beverage.

## 2. Materials and Methods

### 2.1. Materials and Preparation of Infusion

Roasted green tea leaves from the commercial brand ‘Oi Ocha’ (Itoen Ltd., Tokyo, Japan) were sourced from a local supermarket in Matsudo, Japan. For each processed sample, 5 g of tea leaves, with moisture content determined and expressed on a dry basis (d.b.), were placed in a beaker, and hot water at 95 °C was added until the total weight reached 500 g. The mixture was then steeped for 5 min. The resulting infusion was filtered and maintained at 37 °C in a water bath (NTT-20S; Eyela, Tokyo, Japan) to mimic physiological conditions until it was used for simulated in vitro gastrointestinal digestion, which occurred within 30 min.

### 2.2. In Vitro Gastrointestinal Digestion

The in vitro gastrointestinal digestion model, following a two-phase static simulation, was performed twice, adhering to the procedure described by Qin et al. (2022) [16], with slight modifications. A 150 mL portion of tea infusion was placed in a glass reaction vessel, where it was continuously stirred using a magnetic stirrer while the temperature was held at 37 °C via a water bath throughout the entire digestion process. Prior to the gastric phase (labeled as ‘G’), the pH was adjusted to 1.20 using an HCl solution. Afterward, 19 mL of pepsin solution (200 mg of pepsin in 19 mL of 0.1 M HCl) was introduced, and the pH remained at 1.20 ± 0.01 for the duration of the G phase. One hour into the gastric digestion, the process was transitioned to the intestinal phase (denoted as ‘I’). The pH was initially adjusted to 4.0 by adding 1M NaHCO₃ and subsequently raised to 6.8 using an NaOH solution. At this point, 25 mL of intestinal enzyme solution (625 µM pancreatin solution) for the intestinal stage was added. The mixture was maintained under these conditions for another two hours, with the pH kept stable at 6.80 ± 0.01 throughout the I phase. This digestion protocol was referred to as the mimicked digestion under normal digestive conditions (MD). A control experiment was also conducted without the addition of digestive enzymes (WOE) to assess the effect of pH alone on the bioactive properties of the tea infusion. The procedure followed the same steps as the standard digestion. Samples were collected at various stages, as follows: BF, before the digestion process began; G0, at the start of digestion with a pH of 1.2; G1, after 1 h of the gastric phase, with the pH still at 1.2; G1I0, after the gastric phase with the pH adjusted to 6.8; G1I1, following 1 h of the intestinal phase at a pH of 6.8; and G1I2, after 1 h of gastric digestion and 2 h of intestinal digestion, also at a pH of 6.8.

### 2.3. Determination of Bioactive Compounds

The bioactive compounds in the tea samples were assessed by measuring total polyphenol content (TPC) and total flavonoid content (TFC). TPC was quantified using the Fast Blue BB (FBBB) assay, as described in a previous study [16]. Then, 0.1 mL of the sample extract was mixed with 0.01 mL of 0.1% FBBB solution, followed by 0.01 mL of 0.5% NaOH in a 96-well plate. After a 90 min incubation at room temperature, absorbance was measured at 420 nm, and results were expressed as milligrams of gallic acid equivalents per gram dry basis samples.

TFC was determined using a colorimetric assay for the 96-well plate. A 250 µL portion of the diluted sample extract was mixed with 0.01 mL of 5% NaNO_2_, and incubated for 5 min, followed by the addition of 0.01 mL of 10% AlCl_3_ and a 6 min incubation. The reaction was completed by adding 0.06 mL of 1M NaOH, and absorbance was recorded at 520 nm. TFC was expressed as milligrams of catechin equivalents per gram of dry basis. All samples were analyzed for TPC and TFC in triplicate.

### 2.4. Antioxidant Activity

The antioxidant potential of the tea samples was evaluated using the following, several assays: DPPH, ABTS, FRAP, and MIC. DPPH radical scavenging activity was measured following a modified version of the method described by Qin et al. (2022) [16]. A 0.02 mL sample extract was combined with 0.30 mL of 60 µM DPPH solution and incubated in the dark for 30 min. Absorbance was recorded at 520 nm, with results expressed as micromoles of Trolox equivalents per gram of dry basis tea leaves.

For the ABTS test, 0.01 mL sample was mixed with 0.32 mL of diluted ABTS solution. The mixture was incubated for 10 min. After incubation, the absorbance was measured at 740 nm. Results were reported as milligrams of ascorbic acid equivalents per gram of dry basis tea leaves.

Ferric reducing antioxidant power (FRAP) was evaluated by mixing 0.04 mL of the sample extract with 0.26 mL of freshly prepared FRAP reagent (a solution of TPTZ, FeCl_3_·7H_2_O, and acetate buffer). The mixture was incubated at 37 °C for 30 min in the dark, and absorbance was read at 590 nm. FRAP values were expressed as micromoles of FeSO_4_ equivalents per gram of dry basis tea leaves.

Metal ion chelating activity (MIC) was assessed by adding 5 µL of 2 mM FeCl_2_·3H_2_O and 0.01 mL of 5 mM FerroZine^®^ to 0.30 mL of the sample extract. After a 10 min incubation at room temperature, absorbance was measured at 560 nm, and MIC activity was expressed as micromoles of EDTA equivalents per gram of dry basis tea leaves.

### 2.5. Assessment of Bioaccessibility of Bioactive Compounds and Residual Antioxidant Activity

The bioaccessibility and residual antioxidant activity were determined and expressed as percentages (%) using Equations (1) and (2), respectively, as follows:

Bioaccessibility (%) = (BS/BD) × 100(1)

Residual antioxidant activity (%) = (AA/BD) × 100(2)

In Equation (1), BS represents the amount of bioactive compounds quantified at each sampling point during the digestion process. In Equation (2), AA refers to the antioxidant activity measured at each sampling point during digestion, following previously described methods. BD, in both cases, refers to the bioactive compound content and antioxidant activity measured in the initial tea infusion before digestion.

Bioaccessibility refers to the proportion of bioactive compounds that remain accessible for potential absorption at various stages of the digestion process, serving as an indicator of their stability under simulated gastrointestinal conditions. Residual antioxidant activity represents the fraction of antioxidant activity retained at each stage of digestion, expressed as a percentage of the antioxidant activity measured in the initial tea infusion before digestion.

### 2.6. Statistical Analysis

To ensure result accuracy, all experiments were conducted in triplicate. Data analysis was performed using SPSS, Version 26.0 (SPSS Inc., Chicago, IL, USA), with a 95% confidence level (*p* < 0.05). Correlation coefficients for the standard curve were calculated using Microsoft Excel.

## 3. Results and Discussion

### 3.1. Change in Bioactive Compounds During In Vitro Digestion

Table 1 illustrates the changes in total polyphenol content (TPC) and total flavonoid content (TFC) of roasted green tea infusions during different stages of simulated in vitro digestion. Initially, the undigested tea infusion (BF) exhibited the highest levels of TPC (46.49 ± 0.37 mg/g d.b.) and TFC (2.05 ± 0.15 mg/g d.b.), reflecting the high concentration of bioactive compounds before exposure to the digestive process. However, a significant reduction in both TPC and TFC was observed at the G0 stage (before enzyme digestion but after pH adjustment to 1.2). TPC decreased by 2.07-fold to 22.42 ± 0.12 mg/g d.b., while TFC saw a more dramatic 4.27-fold decrease, dropping to 0.48 ± 0.02 mg/g d.b. This drastic reduction highlights the sensitivity of polyphenols and flavonoids to acidic conditions, even in the absence of digestive enzymes. These findings are consistent with the results reported by Ferruzzi and Blakeslee (2007) [17]. They found that polyphenols are prone to oxidation and degradation when exposed to acidic environments. Different types of catechins show varying degrees of stability in alkaline conditions, such as EGCG and EGC breaking down quickly, while EC and ECG are more stable. This shows that catechins react differently depending on the pH level. The sensitivity of polyphenols to pH changes shows how complex their behavior is at different pH levels. It also emphasizes how important the digestive environment is for maintaining polyphenol stability [18].

During the gastric phase (G1), the MD group showed higher retention of both TPC (26.75 ± 1.80 mg/g d.b.) and TFC (0.54 ± 0.02 mg/g d.b.) compared to the WOE group, which exhibited lower values of TPC (21.70 ± 0.07 mg/g d.b.) and TFC (0.38 ± 0.00 mg/g d.b.). It suggests that digestive enzymes play a significant role in stabilizing bioactive compounds under gastric conditions. Digestive enzymes play a critical role in stabilizing bioactive compounds under gastric conditions by enhancing their solubility and retention. This effect is supported by Bohn et al. (2015) [19], who demonstrated that digestive enzymes improved the stability of plant-based compounds during digestion. Additionally, the interaction between tea polyphenols and digestive enzymes through hydrogen bonds and hydrophobic interactions could explain the observed differences. Tea polyphenols binding to enzymes might affect the activity of these enzymes. This is because the structure of tea polyphenols contains hydroxyl and galloyl groups that can interact with the polar parts of the enzymes, changing how they function [20,21].

In the subsequent intestinal digestion phase (G1I0 to G1I2), TPC and TFC remained relatively stable in both groups, with slight variations. At the final digestion stage (G1I2), TPC was 25.13 ± 0.33 mg/g d.b. in the MD group and 23.14 ± 0.71 mg/g d.b. in the WOE group. TFC was also higher in the MD group (1.18 ± 0.02 mg/g d.b.) compared to the WOE group (0.73 ± 0.00 mg/g d.b.). These results emphasize that digestive enzymes not only enhance the release of bioactive compounds but also protect them from further degradation during the intestinal phase. These results emphasize that digestive enzymes enhance the release of bioactive compounds and protect them from further degradation, as also reported by Tagliazucchi et al. (2010) [22]. The various catechin components of green tea have been previously reported to exhibit different stabilities under alkaline pH conditions. In this context, EGCG and EGC are unstable and degrade rapidly. However, the other major catechins, EC and ECG, are more stable under these conditions. In contrast, under acidic pH conditions, all catechins remain stable for several hours [18,23].

### 3.2. Variations in Antioxidant Activity Throughout Simulated Digestion

As natural antioxidants, polyphenols help safeguard biomolecules against oxidative damage, which is linked to their role in disease prevention [24]. Assessing antioxidant activity is often the first step in understanding their biological properties since bioactive compounds must be liberated from the food matrix to exhibit their effects [25]. Antioxidant properties of roasted green tea infusions, including DPPH, ABTS, FRAP, and MIC, were evaluated across different stages of digestion (Table 2). The initial antioxidant activities (BF) of the tea infusions were notably high, with DPPH measured at 66.71 ± 1.37 µmol TE/g d.b., ABTS at 216.94 ± 4.63 mg ascorbic acids/g d.b., FRAP at 236.57 ± 1.80 µmol FeSO_4_/g d.b., and MIC at 7.27 ± 0.11 µmol EDTA/g d.b. These values reflect the robust antioxidant capacity of the roasted tea before any digestion processes. However, upon pH adjustment to 1.2 (G0 stage), a marked reduction in antioxidant activity was observed, with DPPH, ABTS, FRAP, and MIC levels decreasing substantially. These reductions demonstrate the sensitivity of antioxidant compounds to acidic conditions, even before the introduction of digestive enzymes. It is consistent with the significant reduction in polyphenol and flavonoid content at this stage, as noted in Section 3.1, suggesting that the pH adjustment process impacts both bioactive compounds and antioxidant capacity in a similar manner.

During the gastric phase (G1), the MD group exhibited a higher retention of antioxidant activity compared to the WOE group, a trend that parallels the greater stability of polyphenols and flavonoids in the enzyme-treated group noted in Section 3.1, so that digestive enzymes play a key role not only in stabilizing bioactive compounds but also in preserving the associated antioxidant activity. The presence of digestive enzymes helps maintain higher levels of antioxidant compounds in gastric conditions. This finding aligns with the conclusion of Bohn et al. (2015) [19], who stated that digestive enzymes improve the solubility and stability of plant-based compounds. As the digestion progressed through the intestinal phase (from G1I0 to G1I2), antioxidant activity continued to decrease. However, the MD group consistently retained higher antioxidant levels compared to the WOE group. By the end of digestion (G1I2), DPPH, ABTS, and FRAP values dropped more sharply in the WOE group than in the MD group. Even though the MIC values remained more stable in both conditions, the final digestion point showed a higher value than at the beginning of digestion. This result highlights the key role of digestive enzymes in protecting antioxidant compounds from breaking down, especially during the intestinal phase. Since phenolic compounds are known to contribute to antioxidant activity, their retention likely plays a role in the differences in antioxidant capacity observed between the MD and WOE groups. This further confirms that digestive enzymes are important for preserving not only polyphenols and flavonoids but also their related antioxidant effects during digestion.

### 3.3. Bioaccessibility of TPC and TFC and Associated Residual Antioxidant Activity

**Figure 1:**
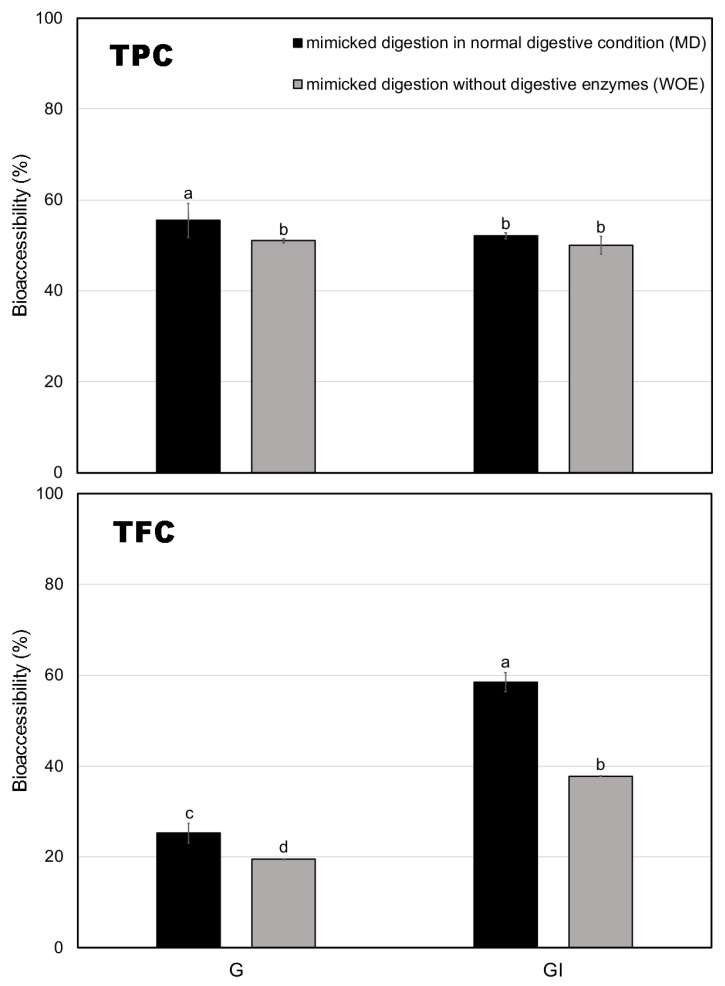
Bioaccessibility (%) of total polyphenol content (TPC) and total flavonoid content (TFC) in roasted green tea infusion during simulated digestion. Bars represent the standard deviation of triplicate determinations. Uppercase letters denote significant differences (*p* < 0.05) between the MD (mimicked digestion) and WOE (without digestive enzymes) treatments within the same compound groups. Lowercase letters indicate significant differences (*p* < 0.05) among different phenolic compound groups within the same digestive condition.

Figure 1 illustrates the bioaccessibility (%) of TPC and TFC under MD and WOE conditions, which demonstrate the substantial impact that digestive enzymes have on the bioaccessibility of total polyphenol content (TPC) and total flavonoid content (TFC). The bioaccessibility of TPC during the G phase reached 56% in the MD group compared to 52% in the WOE group. This slight yet significant increase highlights the role of digestive enzymes in enhancing polyphenol release during the early stages of digestion. Qin et al. (2022) [16] also demonstrated that enzymatic activity plays a crucial role in polyphenol stability and release under gastric conditions, corroborating the trend observed here. As digestion continued through the intestinal phase (GI), TPC bioaccessibility slightly decreased to around 50% in both MD and WOE groups, indicating a stabilization of polyphenol release, as also noted by Hollebeeck et al. (2013) [26], who found a similar plateau effect of polyphenol absorption during digestion. The stability in TPC bioaccessibility at the intestinal stage suggests that a portion of the polyphenols remains resistant to further enzymatic breakdown, highlighting their potential for absorption.

For TFC, the effect of digestive enzymes was even more pronounced. During the G phase, TFC bioaccessibility in the MD group was 25%, compared to only 20% in the WOE group. However, as digestion progressed to the GI phase, the MD group saw a surge in TFC bioaccessibility to nearly 60%, whereas the WOE group increased to only 38%. This significant difference underscores the crucial role of digestive enzymes in flavonoid release, particularly during the intestinal phase. The breakdown of flavonoid glycosides and the release of aglycones through enzymatic hydrolysis may explain the enhanced bioaccessibility observed [27,28]. Nevertheless, it is also important to consider potential limiting factors. Binding with bile salts and interactions with digestive enzymes might lead to the formation of insoluble complexes, partially hindering the release and solubility of flavonoids under intestinal conditions [29,30]. This interplay between enzymatic release and potential bile salt interactions likely determines the overall bioaccessibility of flavonoids. These interactions probably influence the overall bioaccessibility of flavonoids and warrant further investigation.

**Figure 2:**
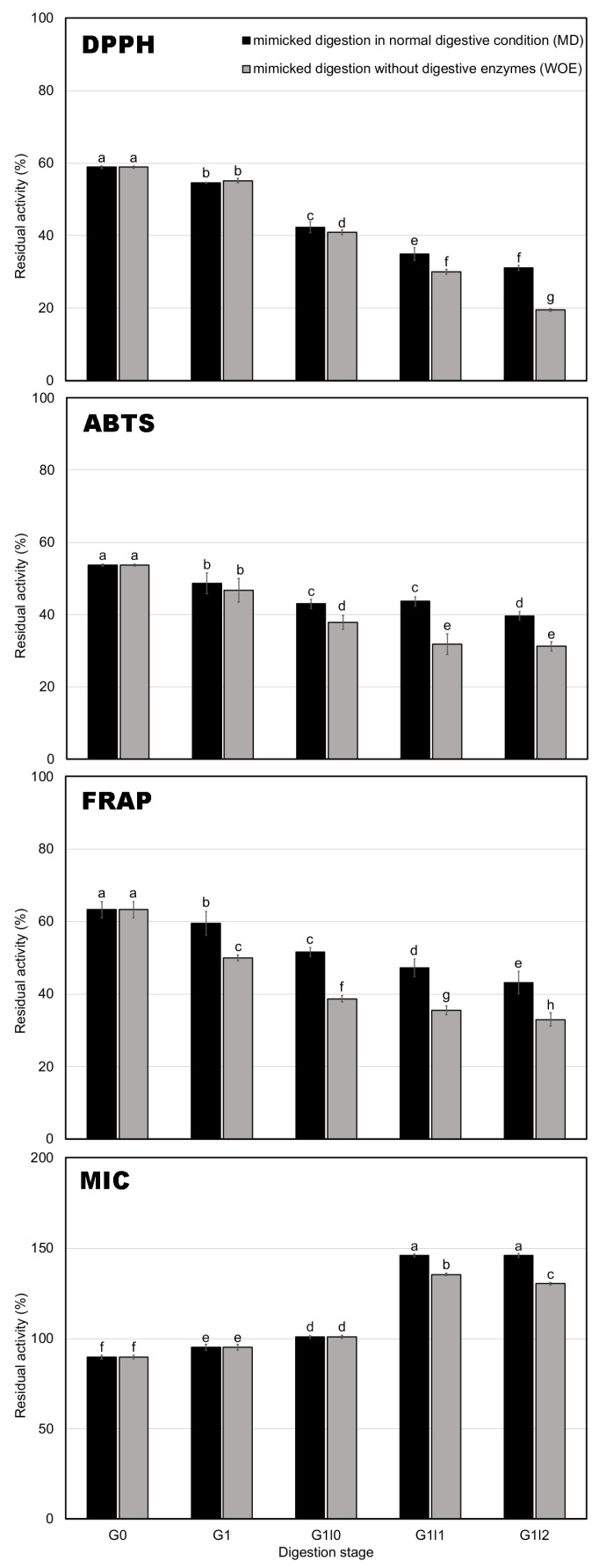
Residual activity of roasted green tea infusion during simulated digestion, evaluated by DPPH, ABTS, FRAP, and MIC assays. Bars represent the standard deviation of triplicate determinations. Lowercase letters indicate significant differences among samples within the digestion stages (G0, G1, G1I0, G1I1, and G1I2) (*p* < 0.05). Uppercase letters denote significant differences between mimicked digestion in MD and WOE within the same digestion stage (*p* < 0.05).

Figure 2 illustrates the changes in residual antioxidant activity (DPPH, ABTS, FRAP, and MIC) during digestion, revealing a clear trend of decline in antioxidant activity across all assays as digestion progressed, particularly in the WOE group. For DPPH activity, the MD group exhibited significantly higher retention compared to the WOE group by the end of the intestinal phase, maintaining 31% of its initial activity compared to only 19% in the WOE group. This 1.63-fold higher retention highlights the role of digestive enzymes in preserving antioxidant potential. A similar trend was observed for ABTS, where the MD group retained 40% of its activity compared to 31% in the WOE group, further underscoring that digestive enzymes helped maintain polyphenol stability and antioxidant activity in tea during digestion, particularly during the intestinal phase [31]. For FRAP activity, the MD group consistently exhibited higher retention (43%) than the WOE group (33%) by the end of digestion. Enzymatic digestion plays a key role in preserving antioxidant activity and the bioaccessibility of polyphenols during gastrointestinal digestion. The increase in free radical scavenging activity is attributed to the deprotonation of the hydroxyl groups on the aromatic rings of phenolic compounds [22]. The transition from the gastric to the intestinal environment may induce structural changes in phenolic molecules, likely due to the ionization of hydroxyl groups. Moreover, some studies have discussed that the antioxidant activity of chemically extracted food compounds may be underestimated because the solvents used for extraction might be less effective in fully extracting polyphenols [32]. Additionally, due to the impact of pH on the racemization of substances, two chiral enantiomers with different reactivities in respective reagents may be formed [33,34,35].

In contrast to the other assays, MIC activity showed an increase during digestion, especially in the intestinal phase, where the MD group exhibited a 1.12-fold higher retention of metal chelation capacity compared to the WOE group. This result suggests that digestive enzymes not only stabilize polyphenolic compounds but also enhance the release of the compounds responsible for metal chelation, as observed in the previous research by Tarko et al. (2020) [36]. The increase in MIC activity, particularly in the MD group, indicates that digestive enzymes play a crucial role in activating bioactive compounds that contribute to the metal chelating capacity of green tea during digestion. Overall, digestive enzymes are essential for preserving the antioxidant capacity of roasted green tea infusions throughout the digestive process, particularly during the intestinal phase, where the bioactive compound release and activation are most critical.

## 4. Conclusions

In this study, the significant influence is shown of digestive enzymes and pH levels on the bioaccessibility of polyphenols, flavonoids, and antioxidant properties in roasted Japanese green tea infusions during simulated in vitro digestion. The conditions of the gastric phase caused significant reductions in total polyphenol content, total flavonoid content, and antioxidant activities. However, the presence of digestive enzymes notably enhanced the release and stability of these bioactive compounds, particularly during the intestinal phase, where TPC and TFC show higher bioaccessibility, as well as antioxidant activity, were consistently higher in MD group samples compared to the WOE group. The findings indicate that digestive enzymes are essential for maintaining the antioxidant capacity of roasted green tea infusions, particularly in the later stages of digestion, where the metal chelating capacity (MIC) also showed increased activity. The results also suggest that enzyme-mediated digestion contributes to the stability and release of bioactive compounds, shedding light on the digestive behavior of roasted green tea. Future studies should explore how different roasting methods and intensities influence the bioaccessibility and functional properties of roasted tea as well as bioactive compound profiles providing deeper insights as a functional beverage. Such research will further clarify the implications of these findings for dietary recommendations and tea product development.

## Figures and Tables

**Table 1:** Total polyphenol content (TPC) and total flavonoid content (TFC) of roasted green tea infusions at various digestion stages.

Digestion Stage	TPC		TFC	
BF	46.49 ± 0.37 AA		2.05 ± 0.15 AA	
G0	22.42 ± 0.12 EC		0.48 ± 0.02 EE	
	MD	WOE	MD	WOE
G1	26.75 ± 1.80 B *	21.70 ± 0.07 D	0.54 ± 0.02 D *	0.38 ± 0.00 F
G1I0	24.30 ± 0.27 C *	21.95 ± 0.33 D	1.23 ± 0.03 B *	0.92 ± 0.02 B
G1I1	23.67 ± 0.27 D *	21.64 ± 0.25 D	1.12 ± 0.03 C *	0.64 ± 0.02 D
G1I2	25.13 ± 0.33 B *	23.14 ± 0.71 B	1.18 ± 0.02 C *	0.73 ± 0.00 C

Different uppercase letters and underlined characters within the same column represent significant differences (*p* < 0.05) across digestion stages for the digested samples under MD and WOE conditions, respectively. An asterisk (*) denotes a significant difference (*p* < 0.05) between the two digestive conditions at the same digestion stage for TPC and TFC.

**Table 2:** Antioxidant properties and pH of roasted green tea infusions at each digestion stage reported as DPPH, ABTS, FRAP, and MIC.

Digestion Stage	DPPH	ABTS	FRAP	MIC
BF	66.71 ± 1.37 AA	216.94 ± 4.63 AA	236.57 ± 1.80 AA	7.27 ± 0.11 BB
G0	34.90 ± 0.03 CC	116.54 ± 0.40 BB	141.93 ± 1.31 BD	6.58 ± 0.06 DD
	MD			
G1	39.25 ± 0.01 B *	105.56 ± 4.38 C *	133.85 ± 6.08 C *	6.85 ± 0.04 C
G1I0	30.56 ± 0.01 D *	93.30 ± 2.38 D *	116.06 ± 0.83 D *	7.27 ± 0.03 B *
G1I1	25.25 ± 0.25 E *	94.84 ± 2.11 D *	106.21 ± 2.41 E *	10.51 ± 0.04 A *
G1I2	22.52 ± 0.16 F *	86.06 ± 1.77 E *	96.31 ± 3.27 F *	10.51 ± 0.03 A *
	WOE			
G1	34.39 ± 0.07 C	102.11 ± 4.43 C	123.40 ± 1.63 C	7.73 ± 0.10 B *
G1I0	24.98 ± 0.07 D	82.53 ± 3.71 D	95.98 ± 2.15 D	5.71 ± 0.03 D
G1I1	18.36 ± 0.17 E	66.49 ± 2.08 E	87.83 ± 0.39 E	9.76 ± 0.00 A
G1I2	11.89 ± 0.03 F	68.23 ± 1.35 E	81.41 ± 0.63 F	9.39 ± 0.02 A

Different uppercase letters and underlined characters within the same column represent significant differences (*p* < 0.05) across digestion stages for the digested samples under MD and WOE conditions, respectively. An asterisk (*) denotes a significant difference (*p* < 0.05) between the two digestive conditions at the same digestion stage for TPC and TFC.

## Data Availability

The original contributions presented in this study are included in the article.
